# Clinical efficacy of fecal microbiota transplantation for patients with small intestinal bacterial overgrowth: a randomized, placebo-controlled clinic study

**DOI:** 10.1186/s12876-021-01630-x

**Published:** 2021-02-06

**Authors:** Fenghua Xu, Ning Li, Chun Wang, Hanyang Xing, Dongfeng Chen, Yanling Wei

**Affiliations:** Department of Gastroenterology, Army Medical Center of PLA affiliated with Army Medical University, No.10 Changjiang Branch Road, Yuzhong District, Chongqing, 400042 China

**Keywords:** Small intestinal bacterial overgrowth, Fecal microbiota transplantation, Therapeutic efficacy, Lactulose breath test

## Abstract

**Background:**

Small intestinal bacterial overgrowth (SIBO) is characterized by the condition that bacteria overgrowth in the small intestine. Fecal microbiota transplantation (FMT) has been applied as an effective tool for reestablishing the structure of gut microbiota. However, whether FMT could be applied as a routine SIBO treatment has not been investigated.

**Methods:**

In this trial, 55 SIBO patients were enrolled. All participants were randomized in two groups, and were given FMT capsule or placebo capsules once a week for 4 consecutive weeks. Measurements including the lactulose hydrogen breath test gastrointestinal symptoms, as well as fecal microbiota diversity were assessed before and after FMT therapy.

**Results:**

Gastrointestinal symptoms significantly improved in SIBO patients after treatment with FMT compared to participants in placebo group. The gut microbiota diversity of FMT group had a significant increase, while placebo group showed none.

**Conclusions:**

This study suggests that applying FMT for patients with SIBO can alleviate gastrointestinal symptoms, indicating that FMT may be a promising and novel therapeutic regimen for SIBO.

Trial registry

This study was retrospectively registered with the Chinese Clinical Trial registry on 2019.7.10 (ID: ChiCTR1900024409, http://www.chictr.org.cn).

## Background

Small intestinal bacterial overgrowth (SIBO) is induced by excessive growth of bacteria in the small intestine and is mainly accompanied with a myriad of gastrointestinal symptoms, including flatulence, dyspepsia, and diarrhea, it is associated with marked adverse effects on quality of life and elevated costs in health care expenditures [1, 2]. SIBO is a common and frequent problem in outpatient practice, especially in irritable bowel syndrome (IBS) patients. Recently, multiple studies have confirmed that SIBO is correlated with irritable bowel syndrome, liver cirrhosis and small intestinal tumors, the presence of SIBO has influences on the progression and prognosis of the abovementioned diseases, both directly and indirectly [3–5]. SIBO patients are often rescripted with antibiotics (i.e., rifaximin, norfloxacin, metronidazole, trimethoprim-sulfamethoxazole, etc.) [6–8], nonetheless, the efficacy of norfloxacin is approximately 37.5% [7], and they cannot be used repeatedly for long periods of time. Beyond that, probiotics can be used to help restore the gut microbial ecosystem, with a curative effect with the antibiotic of 62.8% [9].

Recently, applying fecal microbiota transplantation (FMT) to gastrointestinal diseases has re-emerged and gained increasing attention from medical researchers. For years, FMT has been used as an effective treatment for CDI, as well as other recurrent or refractory gastrointestinal disease [10]. It has been proposed as an effective therapy to restore the gut microbiota barrier by transplanting functional gut microbiota from healthy donors to patients [11]. Compared to probiotics, FMT has more advantages for patients because the transplantation of fecal microbiota is an integral transplantation of intestinal microecology and is “an organ” that humans can truly share, with high safety which would not elicit immune response or rejection like other means of transplanted organ [12]. A published systematic review [13] demonstrated that FMT has the potential to treat ulcerative colitis. El-Salhy [14] reported that FMT is a promising tool for managing IBS.

In our previous work, an encapsulated FMT, named the “intestinal microbiota capsule”, was developed by our team (national invention patent No. 2015103040414) and has been successfully applied in clinical practice. Based on the fact that SIBO is mainly due to the imbalance of intestinal microbiota, we believe that the use of FMT will be of great help in the treatment of SIBO patients. However, the efficacy of FMT treatment for such diseases has not yet been investigated. The present study will first explore the clinical efficacy and safety of FMT for treating SIBO to provide a basis for the clinical application of FMT in the treatment of SIBO.

## Methods

### Study design

Patients were assigned to two groups in this 6-month, randomized, doubled-blind, placebo-controlled study and were subjected to oral-derived FMT capsules or placebo capsules once a week for 4 consecutive weeks. No patients had received antibiotic therapy two months prior to enrollment. All subjects were treated with sixteen capsules once a week for four weeks. Their baseline information of gastrointestinal symptoms was recorded and collected as well as follow-up at 1, 3, and 6 months. Additionally, lactulose hydrogen breath test (LHBT) and CT scan were performed and the fecal microbiota diversity of the patients and donors was analyzed in the FMT and placebo groups at baseline and 6 months.

**Fig. 1:**
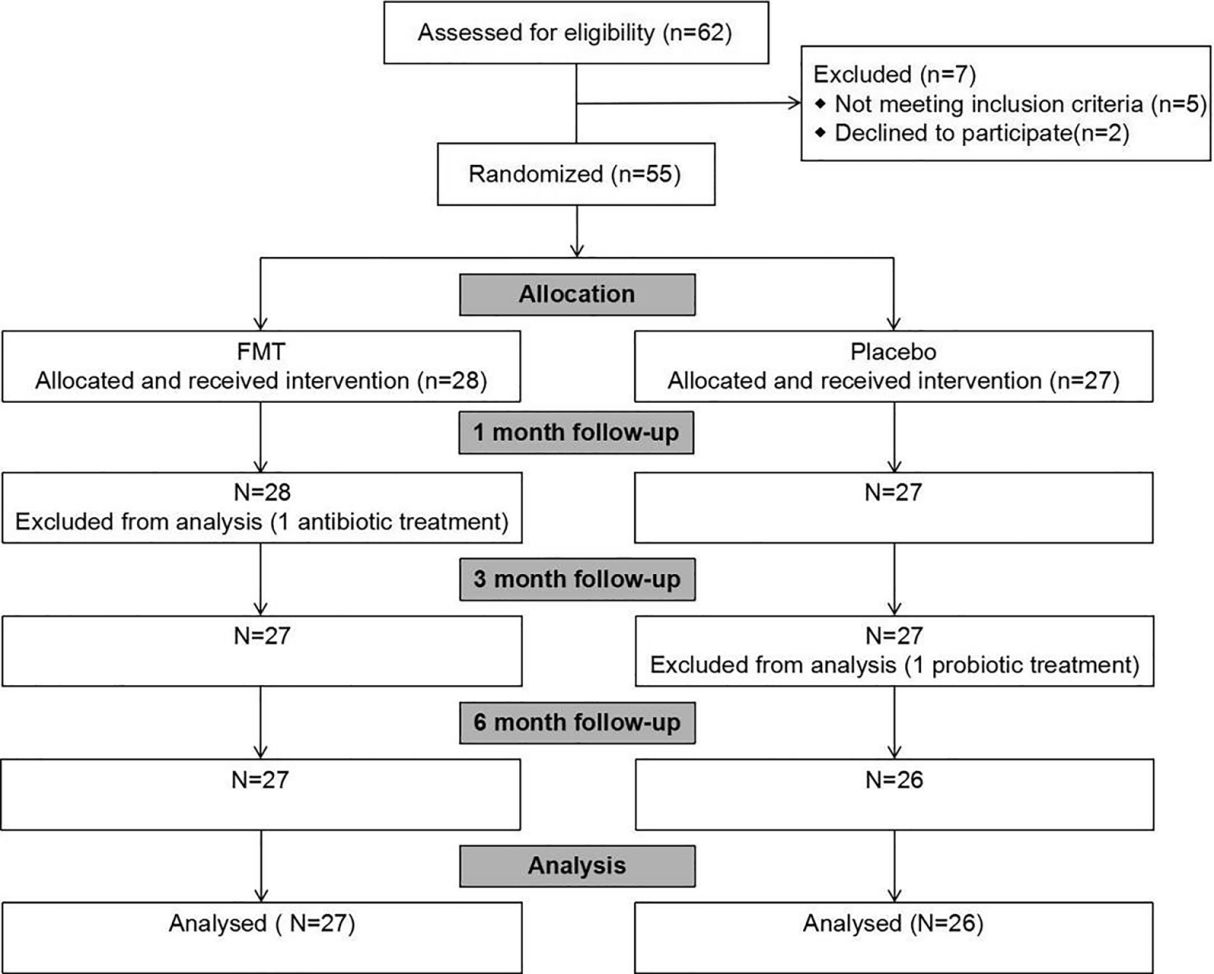
Flow chart of patients included and excluded from analysis. FMT, fecal microbiota transplantation

Our study was reviewed and approved by the Administrative Panel for Medical Research on Human Subjects (the Ethics Committee) of Daping Hospital in Chongqing, China, and informed consent was obtained from all the enrolled subjects, who had full knowledge of the potential risks and benefits. Our study was registered with the Chinese Clinical Trial registry (ID: ChiCTR1900024409, http://www.chictr.org.cn). A flowchart of the procedure is shown in Fig. 1.

### Recruitment of patients and donors

#### Patients

People who were treated in the Department of Gastroenterology, Daping Hospital from October 2016 to October 2018 were prospectively recruited according to the following inclusion and exclusion criteria. All participants have signed informed consent. The diagnosis of SIBO was established as previously reported, by a combination of typical clinical and histological criteria[15, 16]. All enrolled patients had moderate to severe SIBO based on the result of LHBT. Inclusion criteria: Patients were eligible aged between 20 to 60 years old, who had irritable bowel syndrome and were positive in LHBT for SIBO. Medical history, typical clinical symptoms, such as repeatable abdominal distension and percussion of the whole abdomen, or abdominal distension, diarrhea, weight loss, and especially specific signs in CT imaging, can contribute to the diagnosis of this disease. Exclusion criteria: Patients who could not complete the follow-up for at least 60 days after FMT; patients who had used antibiotics, pro-gastrointestinal drugs, intestinal microbicides and laxatives within the last 2 weeks; patients who had received intestinal cleansing or barium enema or who had experienced acute gastroenteritis before colonoscopy in the recent 2 weeks; patients with severe mental illness; pregnant and lactating women; patients who were unable to prepare and complete the test; patients with serious gastrointestinal problems (such as life-threatening intestinal obstruction, perforation and bleeding) that required immediate treatment; patients suffering from ulcerative colitis, Crohn's disease, or celiac disease; cases where the authors were unable to determine whether the inspection results or incomplete data affected the judgment of the results, and cases in which serious adverse events occurred.

#### Donors

Stool donor was selected according to the criteria as follow, based on previously reported studies [16, 17]. Criteria: no history of medication by antibiotic, laxative as well as other associated disease within 3 months; no history of immunomodulator or chemotherapy usage; no history of colorectal polyps, diabetes, no history of IBD, IBS, infectious diseases, morbid obesity; no history of cancer, chronic diarrhea, constipation; no history of allergy, immunocompromised states, metabolic syndrome, chronic fatigue syndrome; no history of major systemic autoimmunity or gastrointestinal surgery, as well as any other situation which might alter intestinal microbiota. Meanwhile, all donors must meet the criteria by laboratory evaluations, including routine blood tests, biochemical tests, Hepatitis evaluations, erythrocyte sedimentation rate, C-reactive protein, human immunodeficiency virus and stool testing. Furthermore, donors were excluded if they have any laboratory abnormalities.

### Production process of FMT capsules

#### Preparation of fecal bacteria liquid

Donors collected feces under close to anaerobic or anaerobic conditions, weighed 100 ~ 150 g samples, and added approximately 250 ml of sterile normal saline for preliminary homogenization to obtain a feces slurry. After removing large particles and fibrous material through a 20-μm nylon filter, the obtained slurry was weighed and further homogenized in a nitrogen bio-engineering kitchen. The feces slurry was filtered step by step through 2.0-, 1.0, 0.4 and 0.1-mm diameter filters and was then filtered through a 0.25-mm filter 2 to 3 times. Samples were centrifuged in a Sorvall SS-34 rotor at 3000 g, 4 ℃ for 5 min. The centrifuged samples were precipitated and then suspended in sterile normal saline with trehalose. The fecal bacteria solution was obtained with its concentration of 60 mg/ml.

#### Preparation of lyophilized powder

First, freeze-drying protectant was added, and the temperature of the fecal bacteria solution was reduced from 25 ℃ to −80 ℃ by a ‘two-step method’. The second step was to lower the temperature at a rate of 3 ~ 5 ℃/min, and the total prefreezing time was 12 ~ 48 h. Then, powder was obtained by vacuum drying for 24 ~ 48 h with a vacuum at 8 ~ 12 pa and a temperature of -40 ~ -60 ℃. The prepared powder was added to an enteric-soluble capsule of 0.9 g/grain at an ultra-clean workbench, and the fecal bacteria capsule was prepared. The capsules were stored at -80 ℃ for later use.

### Lactulose hydrogen breath test (LHBT)

The breath tests were performed according to previously described procedures [18, 19]. Briefly, after 12 h of fasting, a breath test was performed after an oral lactulose load. First, 10 g of lactulose was dissolved in 100 mL of water, and was taken by participants prior to breath test, then breath samples were collected by breath bags (NAMEF, Beijing, China) at baseline and within 120 min (for 20-min time intervals). Hydrogen (H_2_) and methane (CH_4_) concentration were measured by chromatography (NAMEF, Beijing, China).

The result was considered to be positive for SIBO if one of the following criteria was met within 30 to 120 min: (1) H_2_ concentration > 20 ppm over the baseline value within 90 min, (2) CH_4_ concentration > 10 ppm over the baseline value, (3) H_2_ concentration > 20 ppm over the baseline value, and both of the repeated measurements > 20 ppm or (4) CH_4_ concentration > 10 ppm over the baseline value, and both of the repeated measurements > 10 ppm.

The chief endpoints for SIBO were primarily negative results on LHBT and virtually complete relief of symptoms such as bloating and diarrhea. Clinically, there are still a small number of patients with negative results accompanied by clinical symptoms, such as functional diarrhea and functional constipation.

### Gastrointestinal symptom rating scale (GSRS)

The GSRS includes 15 items that assess gastrointestinal symptoms on an interview-based rating scale. The 15 items on the GSRS are presentative for the following five domains: reflux, abdominal pain, ingestion, diarrhea, and constipation. The score for each item in GSRS ranges from 1 to 7, and the results are presented and analyzed as a total syndrome score.

### Microbiota analysis

Fecal samples from all participants were collected at baseline and endpoint of this study, and samples were stored at − 80 °C and sent to the G-BIO company in Hangzhou, China (http://www.igeneseq.com/) for analysis with the use of 16S rRNA-based high-throughput sequencing. Stool samples from patients pre- and post-FMT or pre- and post-placebo treatment were collected and analyzed, as previously reported [15]. Specifically, the 16S V3-V4 regions were amplified based on the following primers: forward primer: CCTACGGGNGGCWGCAG; and reverse primer: GACTACHVGGGTATCTAATCC. Products from each sample were mixed at equal concentration, and were then analyzed by an Illumina MiSeq platform following standard Illumina sequencing protocols. The results of 16S rRNA were analyzed by mothur, UPARSE, and R. Operational taxonomic units (OTUs) were clustered at 97% similarity and filtered by the UPARSE pipeline. Unweighted UniFrac distances were analyzed by mothur, while data visualization was achieved by principal coordinate analysis (PCoA) in R. Significance thresholds were adjusted based on a false discovery rate when making multiple comparisons by the Benjamini–Hochberg approach.

### Statistical analysis

The data were analyzed by SPSS software (20.0). Student's t-test was performed for continuous variables. Two-way ANOVA was used to determine factors associated with a decrease in GSRS score. Otherwise, the Wilcoxon rank-sum test was applied for analyzing differences between groups. P < 0.05 was considered to be significant difference.

## Results

### Enrolled patients were divided into FMT and placebo groups according to their baseline characteristics

Overall, 55 patients were included in this study and were randomized into two groups: the FMT group included 28 patients, while 27 patients in placebo group. The patients in the two groups were statistically comparable (Table 1). No patients dropped out of the study. Other participants were excluded according to the exclusion criteria (Fig. 1). At the end of the study, patients were asked what treatment they were being given, and only 20 patients guessed correctly (12 correctly guessed FMT, and 8 correctly guessed placebo).

**Table 1:** Baseline characteristic of patients

	Overall	FMT	Placebo	P value
N	55	28	27	
Age (mean ± SD)	40.3 ± 6.3	40.9 ± 7.4	39.7 ± 5.2	0.685
Weight (mean ± SD)	60.1 ± 3.9	59.2 ± 3.8	61.0 ± 4.0	0.833
Height (mean ± SD)	1.67 ± 0.07	1.69 ± 0.04	1.65 ± 3.98	0.734
BMI (mean ± SD)	21.7 ± 2.0	20.8 ± 1.6	22.5 ± 2.4	0.906
Male (%)	20(36.4)	10(35.7)	10(37.0)	0.906
Continue use of SIBO medication (%)	33(60.0)	17(60.7)	16(59.3)	0.579
Former tried SIBO medication (%)	42(76.4)	23(82.1)	19(70.4)	0.382
Former tried FODMAP diet (%)	26(47.3)	12(42.9)	14(51.9)	0.652
Born by casarean section (%)	7(12.7)	3(10.7)	4(14.8)	1
PPI (%)	9(16.4)	3(10.7)	6(22.2)	0.974
Antacid (%)	8(14.5)	4(14.3)	4(14.8)	0.523
Bile acid inhibitor (%)	5(9.1)	4(14.3)	1(3.7)	1
Immunosuppressor (%)	2(3.6)	1(3.6)	1(3.7)	1

BMI, body mass index; SIBO, small intestinal bacterial overgrowth; PPI, proton pump inhibitor

### FMT effectively improved gastrointestinal symptoms

**Fig. 2:**
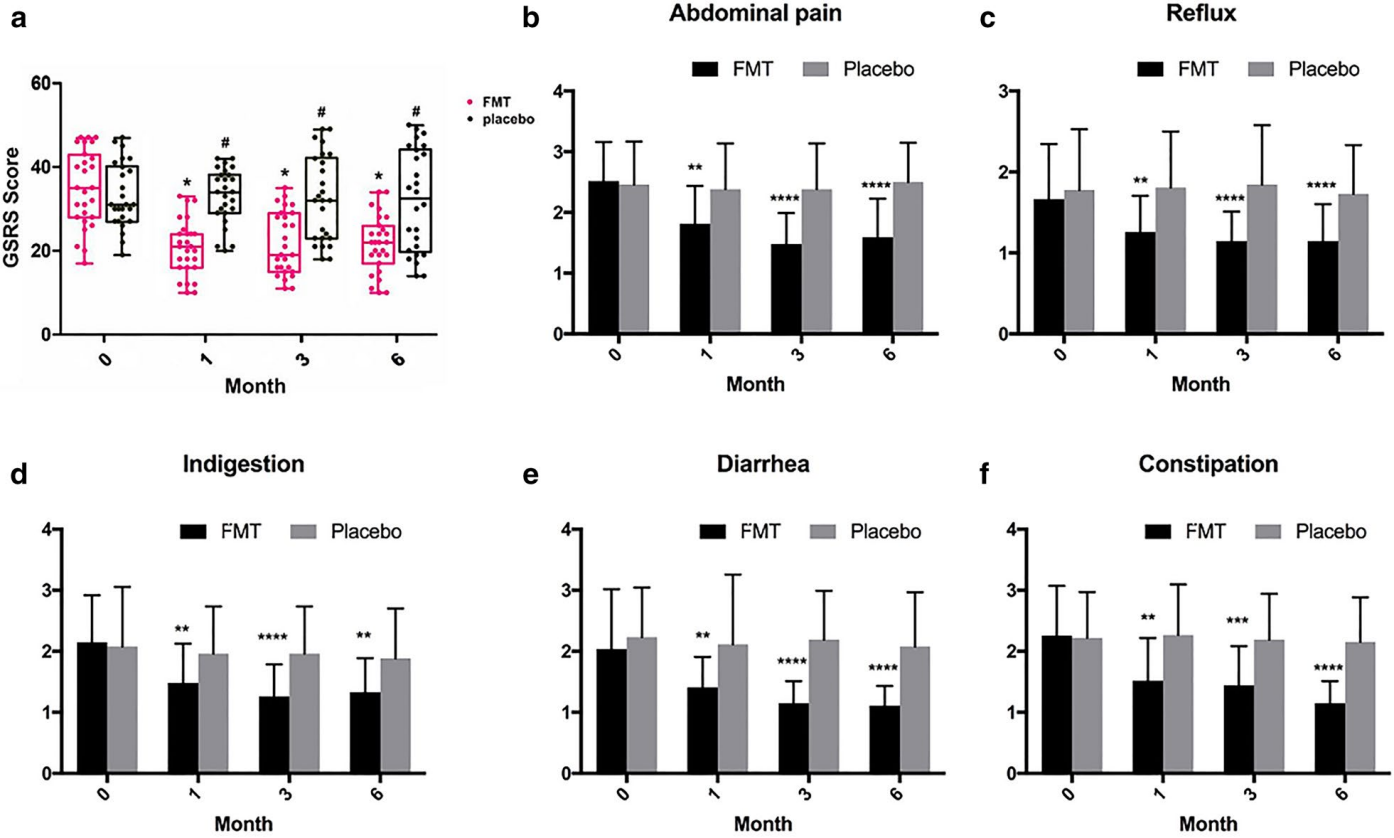
**a** SIBO-GSRS score between groups and their change over time. FMT, fecal microbiota transplantation; GSRS, Gastrointestinal Symptom Rating Scale. *month 1, 3 and 6 vs month 0 in FMT group (P < 0.05); # FMT group vs placebo group in different time-point (P < 0.05). **b**–**f** Average score of abdominal pain, reflux, indigestion, diarrhea and constipation in FMT and placebo group, significant difference was compared between FMT group vs placebo group in different time-point (*: P < 0.05; **: P < 0.01; ***: P < 0.001; ****: P < 0.0001)

The SIBO-GSRS score changes over time at baseline and at 1, 3 and 6 months between the FMT and placebo groups are shown in Fig. 2a. The FMT group had significant changes in the GSRS score during this trial. As shown in Fig. 2, the scores of the end points of the three follow-up visits suggested a significant decrease between baseline and the 1, 3, and 6-month visits, with no significant change in the placebo group, evaluation by GSRS showed that patient in FMT group exhibited improvement in SIBO symptoms compared to placebo group.

### FMT effectively reduced the gas increase caused by SIBO through lactulose hydrogen breath test and CT scan

**Fig. 3:**
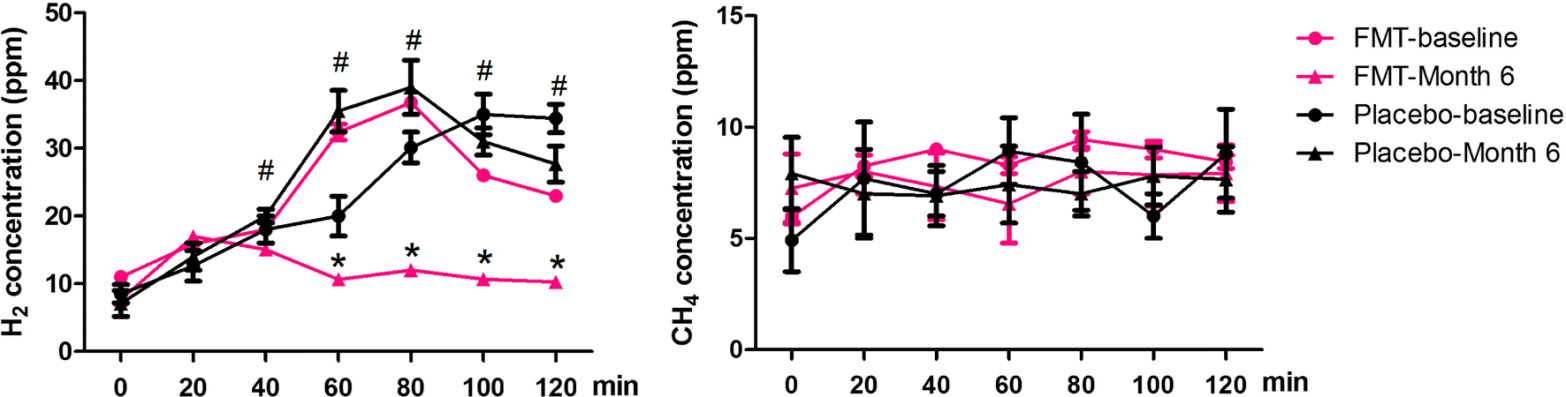
Comparison of LHBT at baseline and after FMT therapy at month 6. **a **The outcomes of LHBT of 53 patients pre- and post-FMT were represented as mean ± SD, respectively. According to the results of LHBT, the H2 concentration were found increased followed with time compared to the results at baseline, but there was no significant change in CH_4_ concentration. *FMT-baseline vs FMT-Month 6 (P < 0.05), # FMT-Month 6 versus placebo-month 6 (P < 0.05)

The changes in concentration of hydrogen and methane in the exhaled gas at different time points were detected by lactulose hydrogen breath test. The H_2_ concentration levels in the exhaled gas of patients were significantly increased at 40, 60, 80, 100 and 120 min, but not the CH_4_ concentrations. In addition, we found that the H_2_ concentration was decreased after FMT treatment compared with the baseline in the FMT group, but not in the placebo group (Fig. 3). The chief endpoints for SIBO were primarily negative results on LHBT and virtually complete relief of symptoms such as bloating and diarrhea.

### FMT changed fecal microbiota diversity and abundance in SIBO patients

**Fig. 4:**
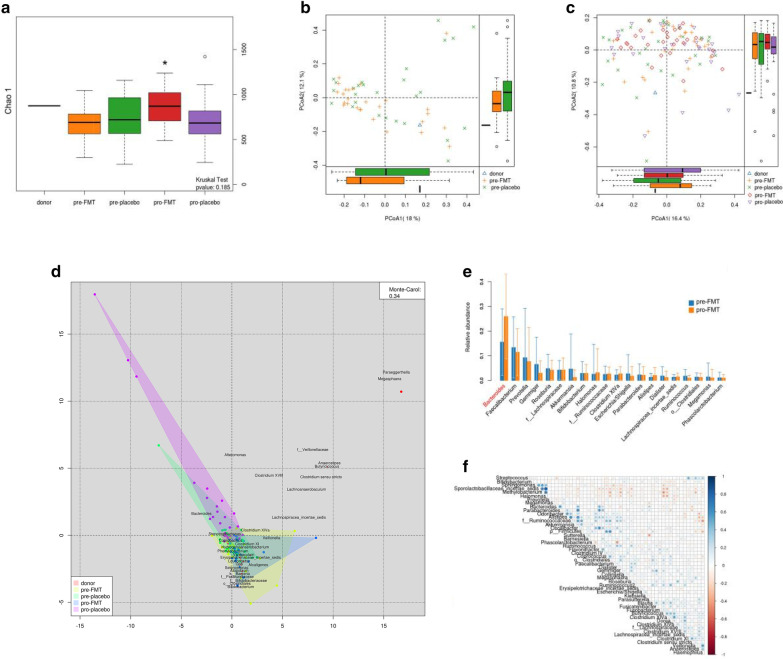
**a** α-diversity of donor, pre-FMT, pre-placebo, pro-FMT, pro-placebo. Black line represented median α-diversities (chao 1), the boxes range from the lower to the upper quartiles. * pre-FMT vs pro-FMT (P < 0.05). **b** β-diversity plots (unweighted UniFrac distance) of donor, pre-FMT, pre-placebo. **c** β-diversity plots (unweighted UniFrac distance) of donor, pre-FMT, pre-placebo, pro-FMT, pro-placebo (pcoa). **d** Enterotype in gut microbiota community of different groups. **e** The difference of gut microbiota at the level of genus in the pre- and pro-FMT group. **f** The plot of correlation gut microbiota at the level of species

Comparisons of fecal microbial diversity and abundance in patients with SIBO before and after FMT were performed by 16S rRNA sequencing analysis. Chao 1 and PCoA indicators, as shown in Fig. 4, were used to analyze the sample differences. Analysis of α-diversity showed the following: (1) donors had higher microbiota biodiversity than patients with SIBO (FMT group and placebo group) at baseline, and (2) the gut microbiota diversity of the participants in FMT group were more similar donors. Analysis of β-diversity (unweighted UniFrac) showed that the microbiota of the donors closed together at the edge of the “cloud” of the microbiota of patients with SIBO. Statistical analysis of unweighted UniFrac confirmed that the microbiota of the FMT recipients were more similar to the microbiota of the donors than to the microbiota of the placebo recipients. Furthermore, the intestinal types of the five groups were not well differentiated, as shown in Fig. 4d.

Additionally, it was found that the composition of fecal microbiota at different levels pre- and post-FMT were different. There was a significant alteration at the genus level in *Bacteroides* abundance before and after FMT therapy.

### No significant side effects were observed in the FMT and placebo groups

According to a previous report, the response rate of CDI patients to FMT is not 100% but can reach more than 80–90% [20]. Because each patient has a different set rate for microbiota after receiving FMT, there will be different response rates and different gastrointestinal reactions. A minority of patients experienced side effects: 1 (3.6%) in the FMT group experienced diarrhea after treatment, and 0 (0%) in the placebo group. Meanwhile, the only one patient in FMT group who experienced a short diarrhea recovered quickly after this adverse event happened. No serious adverse events occurred during this trial.

## Discussion

Fecal microbiota transplantation (FMT) is a technique to transfer the fecal microbiota from healthy donors into patients whose disease is associated altered microbiome, with the goal of restoring gut microbiota for therapeutic outcome [21]. In recent years, FMT has been applied as IBS therapy, with certain achievements [22]. Due to its strong regulating effect on intestinal microecology, FMT may be an effective treatment for diseases related to abnormal intestinal microbiota, such as inflammatory bowel disease, metabolic syndrome, and IBS. Currently, it is widely accepted that FMT is an effective method when antibiotic treatment is ineffective for intractable *C. difficile* infection. The application history of FMT in the therapy of pseudomembranous enteritis and *Clostridium difficile*-associated diarrhea is longer than 50 years, contributing to a very promising therapeutic effect [23, 24]. To date, FMT is currently recommended for use only under clinical guidelines for recurrent *Clostridium difficile*, and other patients, including IBS or IBD, are treated only in clinical trials, including our SIBO patients [4, 25–27]. Staley [28] from the USA proposed that FMT is a straightforward but extremely effective therapeutic treatment for patients with IBD. One of our previous studies preliminarily showed that the prevalence of SIBO in IBS patients was higher than that in healthy controls (51.7% vs. 16.7%, p < 0.001) [29]. As a kind of clinical syndrome caused by intestinal dysbiosis, SIBO may be presentative for the therapeutic efficacy of FMT. Currently, only one patient with SIBO following FMT treatment was reported by Lahtinen and his colleagues [29]. More studies are needed.

Evaluating hydrogen and methane gas in breath still remains as the most convenient, inexpensive, non-invasive tool for SIBO diagnosis. Methane or hydrogen gas would not be produced by human cells in intestine, and these gases would only appear in human breath if the metabolism of carbohydrate residues and absorbed by the gut, and then breathed by the lungs [30]. Based on this principle, when lactulose or glucose is given to a patient with presumed SIBO, the changes in hydrogen and methane concentrations in sequential breath samples will indicate the presence of SIBO [31]. At present, there are many methods for the diagnosis of SIBO. Bacterial culture in small intestinal fluid is the gold standard, but drawing materials is invasive and difficult. LHBT is simple, rapid and non-invasive. In the process of this study, we also carried out the test in strict accordance with the operating specifications to improve the sensitivity and specificity of the test as much as possible. Therefore, LHBT was used as one of the inclusion criteria for patients.

Previous administration of FMT are mainly based on nasogastric tube, nasojejunal tube, gastroscope, colonoscope and retention enema, all of which are invasive and not convenient enough to be widely popularized [32]. Conversely, our self-developed intestinal microbiota capsule is completely orally delivered and is given at an interval of one week, which is non-invasive, convenient, cost-effective and with long-term maintenance for bacterial viability. In 1983, Schwan [33] reported that fecal bacteria liquid treatment in patients showed a curative effect, marking the first time that FMT was used to cure patients. In 2012, Hamilton [34], using standard fecal bacteria for the treatment of 43 patients, with a high power reaching 95%, marked the beginning of standardization of frozen preparation for fecal microbiota, indicating the new route of treatment. In 2013, FMT was one of the top 10 biomedical breakthroughs of the year, which is approved by FDA for the treatment of recurrent CDI. The oral capsule described in this study is acquired after a series of standardized procedures involving the acquisition of a human fecal microbiota, the quantification of the transplanted gut microbiota, the transplantation procedure, and the effective reduction of dead microbiota and has optimally enhanced the separation efficiency of fecal microbiota, promoted the availability of the treatment, and increased the application scope.

In this study, 55 SIBO patients with positive results of LHBT were included, and the symptoms of the 27 patients with SIBO were significantly improved at the end of treatment. The GSRS scores post-FMT were significantly lower than those pre-FMT. FMT did not cause obvious side effects, and only a few patients experienced diarrhea. The results of LHBT showed that FMT effectively improved the clinical symptoms of SIBO patients and transformed LHBT from positive to negative. All results indicate that our oral capsule delivery of FMT is effective for patients with SIBO.

According to previous studies, broad-spectrum antibiotics, especially rifaximin, are generally recommended, resulting in improved symptoms and disease eradication to different extents [6, 35] However, such treatments are not appropriate for all patients with SIBO. In addition, the use of antibiotics is considered to be risky (e.g., antibiotic resistance, serious allergic reaction, and potential fungal infections). On the one hand, gut prokinetic agents are commonly used in the treatment of SIBO because small bowel motility is the most important protective mechanism preventing SIBO [36]. Although this kind of drug can ameliorate symptoms to some extent, it needs to be combined with antibiotics, and patients are required to avoid the use of opioids and other antimotility drugs. On the other hand, the application of an elemental diet can ensure nutritional supplementation and reduce the proliferation of bacteria in the small intestine. However, there have been no results from evidence-based medicine to confirm such a dietary strategy.

The etiology of SIBO is lie on the exceeding growth of bacterial, especially harmful ones, in small intestine, the dysbiosis of small intestinal microbiota would result in typical SIBO symptoms, this paves the possibility of curing SIBO by treatment which could modulate human gut microbiota [37, 38]. Therapies by microbial modulation include probiotics, prebiotics, synbiotics and FMT. Due to the safety, availability and desirable effect, microbial treatments have been widely accepted, and FMT has attracted great interest, in addition, there has been an interest in the use of probiotic and prebiotic agents in the management of SIBO [9]. Although suggested to be beneficial in small studies, the use of probiotic preparations in the management of SIBO remains unproven and requires further study. Probiotic preparations include only a single bacterium or a combination of 2–3 bacteria, which cannot optimally restore the gut microbiota homeostasis either on their own or with prebiotic agents.

In contrast, FMT can help reconstruct the impaired gut microbiota barrier and correct dysbiosis in patients [39], resulting in great therapeutic potential for treating SIBO. According to this prospective clinical trial, improved symptoms and a high negative conversion rate were observed post-FMT compared to pre-FMT, microbiota analysis showing that after FMT treatment, the bio-diversity of gut microbiota in SIBO patients significantly changed, in a manner that more close to the healthy donor. Along with the modulation of gut microbiota, major symptoms of SIBO, such as abdominal pain, reflux, indigestion, diarrhea and constipation ameliorated significantly, due to the microecology restoration effect of FMT. Furthermore, in this study, no serious treatment-related adverse events occurred, suggesting that FMT is an effective and safe therapeutic option and is worth applying in clinical practice. Furthermore, our capsulized FMT solved the clinical problems associated with the long-term maintenance of fresh fecal microbiota, repeated transplantation and invasive procedures, which is of great significance for optimizing the traditional FMT clinical strategy. Furthermore, at different levels, changes in the proportions of fecal microbiota generally approached those of the donor, demonstrating that the oral-delivered FMT has great potential to restore the homeostasis of the gut microbiota. The changes in microbiota composition observed in this study could also heighten our confidence in generalizing the application of FMT therapy.

However, the limitations of this work also need to be taken into consideration. The analysis of the results in the gut microbiota was not ideal. The possible reason may be the sole donor included in this trial. It is possible that more donors for a fecal mix would be preferable. In our report, it was found that Bacteroides were significantly increased in patients post-FMT compared to pre-FMT, implying that FMT therapy could effectively change the composition of the gut microbiota and restore the colonization of beneficial bacteria. However, because of the limited sample size, changes in other beneficial bacteria were not demonstrated in the current research, and further study is needed.

## Conclusions

In summary, this study suggested that the encapsulated formulation of FMT, as a novel treatment for SIBO, is effective and safe, and has promising potential for further translation from bench to beside. Also, the limitation of this study suggested that clinical trials involving larger patient samples in randomized controlled trials with longer follow-up are warranted.

## Data Availability

The data generated by and used in the study is available from the corresponding author upon reasonable request.

## Abbreviations

SIBO: Small intestinal bacterial overgrowth
FMT: Fecal microbiota transplantation
CT: Computed tomography
IBS: Irritable bowel syndrome
LHBT: Lactulose hydrogen breath test
IBD: Inflammatory bowel disease
GSRS: Gastrointestinal Symptom Rating Scale
OTUs: Operational taxonomic units
PCoA: Principal coordinate analysis
SPSS: Statistical Product and Service Solutions
ANOVA: Analysis of Variance
BMI: Body mass index
PPI: Proton pump inhibitor
FDA: Food and Drug Administration
